# The Monitoring and Evaluation of a Multicountry Surveillance Study, the Severe Typhoid Fever in Africa Program

**DOI:** 10.1093/cid/ciz597

**Published:** 2019-10-30

**Authors:** Ondari D Mogeni, Ligia María Cruz Espinoza, Justin Im, Ursula Panzner, Trevor Toy, Gi Deok Pak, Andrea Haselbeck, Enusa Ramani, Heidi Schütt-Gerowitt, Jan Jacobs, Octavie Lunguya Metila, Oluwafemi J Adewusi, Iruka N Okeke, Veronica I Ogunleye, Ellis Owusu-Dabo, Raphaël Rakotozandrindrainy, Abdramane Bassiahi Soura, Mekonnen Teferi, Keriann Conway Roy, William Macwright, Robert F Breiman, Jerome H Kim, Vittal Mogasale, Stephen Baker, Se Eun Park, Florian Marks

**Affiliations:** 1 International Vaccine Institute, Seoul, Republic of Korea; 2 Institute of Medical Microbiology, University of Cologne, Germany; 3 Department of Microbiology and Immunology, KU Leuven, Antwerp, Belgium; 4 Department of Clinical Sciences, Institute of Tropical Medicine, Antwerp, Belgium; 5 Institut National de Recherche Biomédicale, Democratic Republic of Congo; 6 Service de Microbiologie, Cliniques Universitaires de Kinshasa, Democratic Republic of Congo; 7 College of Medicine, University of Ibadan, Nigeria; 8 Faculty of Pharmacy, University of Ibadan, Nigeria; 9 School of Public Health, Kwame Nkrumah University of Science and Technology, Kumasi, Ghana; 10 University of Antananarivo, Madagascar; 11 Institut Supérieur des Sciences de la Population, University of Ouagadougou, Burkina Faso; 12 Armauer Hansen Research Institute, ALERT Campus, Addis Ababa, Ethiopia; 13 Global Health Institute, Emory University, Atlanta, Georgia; 14 Department of Medicine, Cambridge University, United Kingdom; 15 Hospital for Tropical Diseases, Wellcome Trust Major Overseas Programme, Oxford University, Clinical Research Unit, Ho Chi Minh City, Vietnam

**Keywords:** monitoring, *Salmonella*, Africa, epidemiology, multi-country

## Abstract

**Background:**

There is limited information on the best practices for monitoring multicountry epidemiological studies. Here, we describe the monitoring and evaluation procedures created for the multicountry Severe Typhoid Fever in Africa (SETA) study.

**Methods:**

Elements from the US Food and Drug Administration (FDA) and European Centre for Disease Prevention and Control (ECDC) recommendations on monitoring clinical trials and data quality, respectively were applied in the development of the SETA monitoring plan. The SETA core activities as well as the key data and activities required for the delivery of SETA outcomes were identified. With this information, a list of key monitorable indicators was developed using on-site and centralized monitoring methods, and a dedicated monitoring team was formed. The core activities were monitored on-site in each country at least twice per year and the SETA databases were monitored centrally as a collaborative effort between the International Vaccine Institute and study sites. Monthly reports were generated for key indicators and used to guide risk-based monitoring specific for each country.

**Results:**

Preliminary results show that monitoring activities have increased compliance with protocol and standard operating procedures. A reduction in blood culture contamination following monitoring field visits in two of the SETA countries are preliminary results of the impact of monitoring activities.

**Conclusions:**

Current monitoring recommendations applicable to clinical trials and routine surveillance systems can be adapted for monitoring epidemiological studies. Continued monitoring efforts ensure that the procedures are harmonized across sites. Flexibility, ongoing feedback, and team participation yield sustainable solutions.

Vaccination is one of the most cost-effective public health interventions for preventing infectious diseases, and in turn, minimizing lost wages and productivity losses due to illness and death [1]. In any setting, these benefits can only be realized and evaluated when burden of disease data are available and can be used to guide vaccine introduction and evaluation [1]. A lack of disease burden data limits public health capacity to guide these interventions and to develop the necessary policy to support the process. Therefore, research studies are critical for generating data to inform the creation and rollout of public health interventions at the country, regional, and global levels [2, 3].

A systematic multicountry research approach offers the advantage of generating standardized burden of disease data that allow for comparison between countries and can be (cautiously) generalized to a region. Regulated clinical trials, interventional studies that will provide data in support of licensure of vaccines or drugs, follow an international set of guidelines known as Good Clinical Practice (GCP) [4]. Epidemiological studies, however, are not subject to GCP requirements, but ensure data quality. Ensuring human safety, data quality and compatibility in multicountry studies is essential for the correct interpretation and applicability of the data. Standardization of the study protocol and standard operating procedures (SOPs) define a pathway for multicountry comparability. Monitoring during study implementation ensures the protection of human subjects and the correct application of such protocols and SOPs while identifying gaps and opportunities for improvement in quality.

While monitoring and evaluation are mandatory in clinical trials [5, 6], with the US Food and Drug Administration (FDA) recommending a risk-based monitoring strategy [7], there is limited reported experience in monitoring best practices in epidemiological studies. The European Centre for Disease Control and Prevention (ECDC) and the World Health Organization (WHO) have outlined strategies for monitoring and evaluation [8, 9]; these guidelines, however, are largely for surveillance in routine healthcare systems and focus on data quality monitoring. Here, we present the monitoring and evaluation procedures implemented in the multicountry Severe Typhoid Fever in Africa (SETA) program [10], adapted from the FDA and the ECDC recommendations on monitoring clinical trials and data quality, respectively. We also present preliminary data of the monitoring impact from two of the six countries participating in SETA.

## METHODS

### Description of the SETA Program

The SETA [10] program is a study that was implemented in six countries in sub-Saharan Africa: Burkina Faso, Democratic Republic of Congo, Ethiopia, Ghana, Madagascar, and Nigeria. The aim of the study was to investigate the burden, severity, and long-term sequelae of typhoid fever and other invasive *Salmonella* infections with the purpose of contributing data for the decision-making process of typhoid vaccine introduction in the African region. The protocol of the study has been published elsewhere [10].

The SETA surveillance system was established to identify invasive *Salmonella* infections among patients of all ages with prolonged fever in the defined catchment areas (Table 1). Patients meeting the inclusion criteria (Table 1) were enrolled into the study upon providing voluntary written informed consent. Blood, an oropharyngeal swab, a stool sample, a urine sample, and in cases of intestinal perforation, tissue sample(s), were taken from each enrolled patient. Samples were transported to the study laboratory for processing, analysis/testing, and storage. Confirmation of invasive bacterial disease was accomplished by blood culture. Patients testing positive for any *Salmonella* (cases) or those with intestinal perforation (special cases) were enrolled into long-term follow-up studies. In these long-term follow-up studies, two or three immediate household contacts and between two and four neighborhood controls meeting the inclusion criteria (Table 1) were identified, consented, and followed up over a period of 360 days. During the follow-up period, cost of illness (conducted in four of the six countries), long-term disease outcomes, and immune responses of cases were assessed.

**Table 1.** Description of the Severe Typhoid in Africa Program

	Key Elements of SETA Surveillance Program
Objectives & outcomes	1. To estimate the burden and severity of invasive *Salmonella* infections a. Population-based adjusted incidence of invasive *Salmonella* infections b. Incidence of hospital- and community-based complications c. Mortality rate and long-term sequelae of *Salmonella* Typhi infections over 1-year follow-up d. Prevalence of antimicrobial resistance among *Salmonella* isolates identified
	2. To assess the immune response to natural TF, PF, and iNTS infections over a 1-year follow-up period a. Assessment of the magnitude and duration of the immune response to TF, PF, and iNTS after natural infection b. Identification and validation of immunological markers associated with the development of *Salmonella* Typhi, *Salmonella* Paratyphi, and iNTS carriage
	3. To estimate the prevalence of *Salmonella*Typhi, *Salmonella* Paratyphi, and NTS carriers among immediate household members of positive *Salmonella* cases a. Prevalence of *Salmonella* Typhi, *Salmonella* Paratyphi, and NTS carriers among the immediate household members of positive cases b. Immunological characterization of *Salmonella* Typhi, *Salmonella* Paratyphi, and NTS carriers c. Description of circulating *Salmonella* Typhi, *Salmonella* Paratyphi, and NTS isolates in the community and blood culture–confirmed cases
	4. To estimate public and private expenditures for treatment and productivity loss associated with illness due to TF, PF, and iNTS infections^a^ a. Calculation of per-case direct and indirect cost of illness for TF, PF, and iNTS categorized by payer and stratified by age and type of service used
	5. To estimate the effects of invasive salmonellosis on the quality of life of patients and the subsequent disease-related family and societal burdens over a 1-year follow-up period^a^ a. Assessment of the quality of life of patients affected with invasive salmonellosis compared to a control group over a 1-year follow-up period
	6. To validate a new rtPCR assay for the diagnosis of invasive *Salmonella* infections in selected SETA sites^a^ a. Validation of rtPCR assay for the diagnosis of invasive *Salmonella* infections
Inclusion criteria	1. Suspected *Salmonella* disease in tertiary health facilities^b^ enrolling for SETA: Fever reported for ≥3 consecutive days within the last 7 days in patients living in the defined catchment area OR Clinically suspected typhoid fever patients living in the defined catchment area OR Blood culture positive for *Salmonella* Typhi/iNTS/*Salmonella* Paratyphi (outside SETA) in patients living in the defined catchment area OR Pathognomonic gastrointestinal perforations (ie, clinically diagnosed typhoid fever gastrointestinal perforation), even in the absence of laboratory confirmation, in patients living in and outside the defined catchment area (special cases) AND Informed consent signed
	2. Suspected *Salmonella* disease in primary and secondary health facilities^b^ enrolling for SETA: Patients living in the defined catchment area presenting to the healthcare facility with objective fever of ≥38°C tympanic and/or ≥37.5°C axillary OR Fever reported for ≥3 consecutive days within the last 7 days in patients living in the defined catchment area OR Clinically suspected typhoid fever patients living in the defined catchment area OR Blood culture positive for *Salmonella* Typhi/iNTS/*Salmonella* Paratyphi (outside SETA) in patients living in the defined catchment area OR Pathognomonic gastrointestinal perforations (ie, clinically diagnosed typhoid fever gastrointestinal perforation), even in the absence of laboratory confirmation, in patients living in and outside the defined catchment area (special cases) AND Informed consent signed
	3. Neighborhood Controls (NCs)Age (±5 years), sex and residency (neighborhood) matched to *Salmonella* Typhi/*Salmonella* Paratyphi/iNTS disease and special cases ANDNo subjective or objective fever at any point within 28 days prior to enrollmentANDNo subjective or objective fever on the date of case enrollment (the “focal time”) ANDInformed consent form signed
	4. Household Contacts (HCs)Immediate household contacts of *Salmonella* Typhi/*Salmonella* Paratyphi/iNTS disease and special cases Priority should be given to: (1) Individual(s) who prepares food for the case;(2) Individual(s) closest in age to the case;(3) Individual(s) who spends the most time with the caseAlternative household contacts should be enrolled if individuals meeting the above priority do not wish to participate or are not identified.ANDInformed consent form signed
Data source(s)	1. Healthcare facilities participating in SETA
	2. Follow-up activities in cases, controls, and contacts identified during surveillance activities
	3. Laboratories participating in SETA (on site and reference laboratories)
Population under surveillance	Burkina Faso, Ethiopia, Ghana, Democratic Republic of Congo, Madagascar, and Nigeria
Surveillance sites	Burkina Faso: Ouagadougou; Kossodo Ghana: Kumasi metropolitan area and Asante Akim North District Madagascar: Imerintsiatosika and Antananarivo Ethiopia: Adama, Addis Ababa, and Welayita Sodo DRC: Kisantu Nigeria: Ibadan
Type of surveillance	Passive, sentinel, voluntary
Information to be reported	Case-based information collected on a daily basis from different data sources
Reporting format	Electronic (Nigeria); paper-based (Burkina Faso, DRC, Ethiopia, Ghana, Madagascar)
Data entry	Electronic tablet (SETA Collect app for Android) Manual data entry
Database architecture	Data collected electronically is held in the IVI server hosted in Seoul, South Korea. Data entered from paper-based forms are stored on local computers and sent to IVI monthly.

Abbreviations: iNTS, invasive nontyphoidal *Salmonella*; IVI, International Vaccine Institute; PF, paratyphoid fever; rtPCR, real-time polymerase chain reaction; SETA, Severe Typhoid Fever in Africa; TF, Typhoid fever.

^a^Objectives not applicable to all SETA countries with a protocol different from the main SETA protocol and standard operating procedures. Monitoring procedures related to these objectives are excluded from this manuscript.

^b^Any suspected case must sign an informed consent form to be included in the study.

### Overview of FDA and ECDC Recommendations for Monitoring

**Figure 1.**
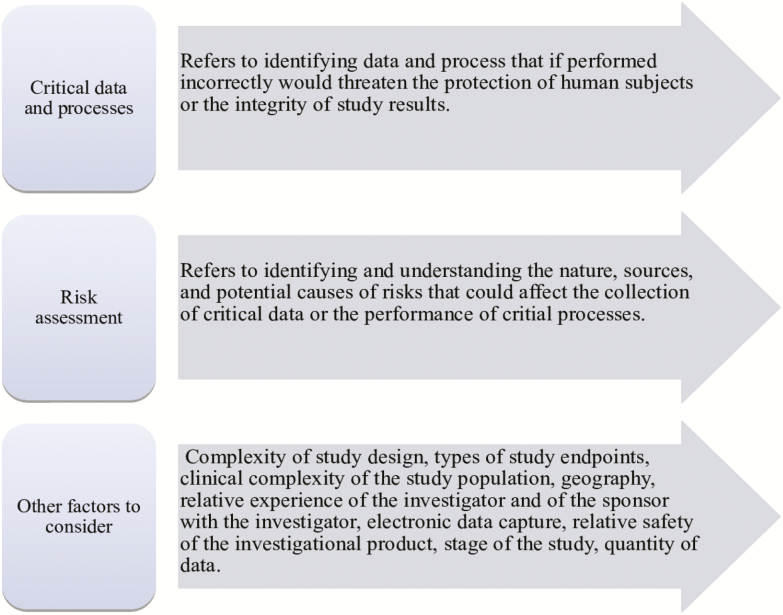
Aspects to consider when developing a monitoring plan tailored to study needs.

The FDA recommends developing a monitoring plan that is tailored to the needs of a study. As such, no single approach to monitoring is necessary for every study, and a mix of centralized and on-site monitoring practices can be part of a risk-based plan [7]. Overall, critical data and processes should be identified and a risk assessment should be conducted before developing a monitoring plan that focuses on the important and likely risk to data and processes (Figure 1).

The ECDC recommends monitoring the surveillance system and data quality [8]. To achieve this, it uses an approach that is attribute-oriented focusing on the surveillance system’s attributes such as data completeness and validity, timeliness, usefulness, representativeness, simplicity, flexibility, and so forth. To quantitatively measure these attributes, ECDC encourages the development of data quality indicators (e.g., for completeness, completeness of data reported; for validity, proportion of cases complying with case definition) and settings targets for monitoring. These indicators could be prioritized and be part of a data quality monitoring report.

### Development of SETA Monitoring Plan

Elements from both the FDA and ECDC recommendations were taken into account and applied in the development of the SETA monitoring plan.

**Figure 2.**
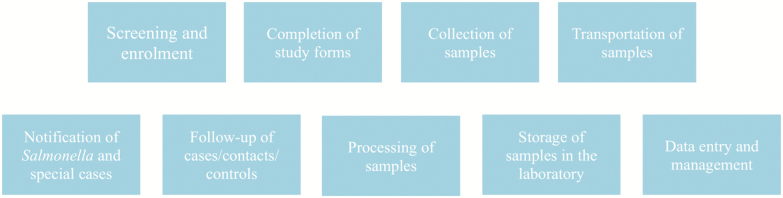
Core activities in the SETA program.

Using the FDA framework, we identified the SETA critical processes that if inaccurate, not performed, or performed incorrectly would affect the delivery of outcomes from the three first SETA objectives (Table 1). We referred to these processes as SETA core activities (Figure 2). They include patient screening and enrollment, study form completion, sample collection, sample transportation, *Salmonella* cases/special cases notification, follow-up, storage and sample shipment, sample processing, data capturing, data entry, and data management.

From the risk assessment, we identified the type of data to be collected to deliver the outcomes from the three first SETA objectives (Table 1) and the specific core activities/processes required to collect these data. Using this information, a list of key indicators was developed to be monitored using on-site and centralized monitoring methods. Table 2 presents the list of data indicators monitored using a centralized method and Table 3 shows the list of processes or core activities monitored using on-site monitoring. Additionally, a team was created to dedicate fully to monitoring activities on-site. SETA databases were used in centralized monitoring at the International Vaccine Institute (IVI).

**Table 2.** Severe Typhoid in Africa Objective-specific Monitoring Indicators

Objective 1: To estimate the burden and severity of invasive *Salmonella* infections
(1) % of screened patients eligible for enrollment (2) % of eligible patients enrolled (3) % of patients enrolled with a blood culture done (4) % of contamination in blood cultures done (5) % of patients with any bacteremia reported (6) % of patients^a^ reported with bacteremia with ST/SP/iNTS isolates (7) % of ST/SP/iNTS isolates stored (8) % of complications from ST/SP cases (9) % of ST/SP cases reported as death (10) % of iNTS cases reported as death (11) % of SC reported as death (12) % of *Salmonella* BC isolates with AMR^b^ performed (13) % of cases with plasma and cells stored from enrollment (14) % of cases with stool stored from enrollment (15) % of iNTS cases with swabs stored from enrollment (16) % of SC with tissue stored from surgery
Objective 2: To assess the immune response to natural TF, PF, and iNTS infection over a 1-year follow-up period
Objective 3: To estimate the prevalence of *Salmonella* Typhi, Salmonella Paratyphi, and NTS carriers among immediate household members of positive *Salmonella* cases
(1)% cases with V1–V5 completed (2)% of ST/SP/iNTS cases with plasma stored from V1–V5 (3)% of ST/SP/iNTS cases with blood cells stored from V1–V5 (4)% of iNTS cases with swabs stored from V1–V5 (5)% of cases with stool analyzed from V1–V5 (6)% of cases with stool stored from V1–V5 (7)% of cases with at least 2 NCs enrolled (8)% of cases with at least 2 HCs enrolled (9)% of NCs with V1–V2 completed (10) % of HCs with V1–V2 completed (11) % of NCs with blood cells stored from recruitment, V1–V2 (12) % of NCs with plasma stored from recruitment, V1–V2 (13) % of NCs with stool analyzed from recruitment, V1–V2 (14) % of NCs with stool stored from recruitment, V1–V2 (15) % of NCs-iNTS with swabs stored from recruitment, V1–V2 (16) % of HCs with blood cells stored from recruitment, V1–V2 (17) % of HCs with plasma stored from recruitment, V1–V2 (18) % of HCs with stool analyzed from recruitment, V1–V2 (19) % of HCs with stool stored from recruitment, V1–V2 (20) % of HCs-iNTS with swabs stored from recruitment, V1–V2 (21) % of cases lost to follow-up (22) % of NCs lost to follow-up (23) % of HCs lost to follow-up

Abbreviations: AMR, antimicrobial resistance; BC, blood culture; HC, healthy control; iNTS, invasive non-typhoidal *Salmonella*; NC, neighborhood control; NTS, non-typhoidal *Salmonella*; SC, stool culture; SP, *Salmonella* Paratyphi; ST, *Salmonella* Typhi; V, visit.

^a^Cases: patients with blood culture–confirmed invasive *Salmonella* infection.

bAMR done for ampicillin, amoxicillin-clavulanic acid, amoxicillin, nalidixic acid, chloramphenicol, cotrimoxazole, ceftriaxone, and ciprofloxacin.

**Table 3.** List of Procedures to Monitor and Observe in the Field

1. Procedures at the HCF
Screening of patients
• Screening of patients visiting the HCF • Documentation of screening procedures • Checking of inclusion criteria • Approach/invitation to participate • Refusals
Enrollment of patients
• Enrollment of patients • Informed consent process ◦ Individual given time to read/hear the ICF content ◦ Individual given time to ask questions ◦ Individual completing and signing the ICF ◦ Individual provided copy of the ICF ◦ Storage of ICF • Completion of study forms • Collection of samples ◦ Phlebotomy technique ◦ Instructions given to participants to collect urine and stool ◦ Distribution of blood collected into different vials according to type of vial and site ◦ Document time/days to collect stool/urine samples ◦ Collection of swab samples • Documenting results and costs of tests not required by the study
Review of completed and stored study forms
• Place of storage of completed study forms • ICF collected between the previous and the current field visits and check completion • Review of completed forms (5%) and check errors, corrections, and missing data
Management of samples
• Storage location and temperature of blood culture bottles before use • Storage conditions (temperature and place of storage) of samples collected before transportation to the laboratory • Documentation of sample transportation (variables recorded) • Processing of samples at the sites • Procedures to transport samples (time, temperature, place of storage) • Procedures to receive and process samples at the designated laboratory • Reporting of results strategy, specially notification of *Salmonella* cases • Laboratory logbook
Follow-up of patients under observation
• Organization and strategy to follow up hospitalized patients • Daily documentation of health status until patient discharge • Daily documentation of tests performed after enrollment until patient discharge • Completion of forms during hospitalization and at the moment of discharge • Collection of samples in patients requiring surgery • Strategy to identify special cases
2. Procedures at enrollment of HCs and NCs and during follow-up visits of cases/HCs/NCs
Screening and enrollment of NCs and HCs
NCs
• Organization of visits to the community to screen and recruit NCs • Procedure to identify eligible households for NC screening and enrollment • Procedure to identify eligible controls within the selected household (case-control age matching) • Cases with 4 NCs enrolled within the 10 days of case enrollment • Refusals (frequency and reasons) • Capturing of GPS coordinates
HCs
• Organization of visits to the house of the case to recruit HCs • Procedure to identify the house of the case • Procedure to identify eligible contact within the house of the case (contact profile priority) • Cases with 3 HCs enrolled within the 10 days of case enrollment • Refusals (frequency and reasons)
NCs + HCs
• Informed consent process (procedures described in the enrollment of patients’ section) • Completion of study forms • Sample collection (procedures described in enrollment of patients’ section)
3. Other field procedures to monitor
Activities in the laboratory
• Sample reception at the laboratory and storage conditions before processing • Logbook for SETA samples • Documentation of blood culture bottles weighting before and after blood sample collection • Fridge, freezer, and incubator log sheets. Are they kept up to date? • Storage of different SETA samples (organization, logbooks/devices registering the temperature of freezers) • Completion of laboratory forms • Presence of quality procedure guidelines/SOPs • Procedure to notify HCF of *Salmonella*-positive cases and other test results (communication between laboratory and HCF)
Data entry and management
• Data capturing (paper, electronic) • Data entry (when applicable) • Procedures and time taken to correct queries • Use and challenges of SETA data entry system • The time to enter enrollment information (if paper-based system used) • Reporting of data to IVI (organization and frequency)

Abbreviations: GPS, Global Positioning System; HC, healthy control; HCF, healthcare facility; ICF, informed consent form; IVI, International Vaccine Institute; NC, neighborhood control; SETA, Severe Typhoid Fever in Africa; SOP, standard operating procedure.

### On-site Monitoring of Core Activities

The aim of monitoring of core activities in the field was to carefully observe the adherence of the study procedures during visits by the IVI monitoring team (Table 3). The monitoring of core activities in each field visit was organized into three major areas: (1) healthcare facilities, (2) community, and (3) laboratory and data management.

#### Healthcare Facilities

The procedures to identify eligible patients according to the study protocol included obtaining voluntary informed consent, enrollment, collection of samples, and documentation of observations at the healthcare facility. These procedures took place at the entry points within the specified healthcare facility in outpatient/inpatient departments. Where the source documents were patient charts, they were checked to corroborate the information written in the study case report forms. To ensure harmonization of patient identification and recruitment procedures across all sites and within enrollment points, a screening and recruitment log (Supplementary Figure 1) was developed and deployed at each site’s entry point.

#### Community

The procedures observed in the field included enrollment of household contacts, neighborhood controls, and follow-up visits of the cases, contacts, and controls. At each follow-up visit, the cases, household contacts, and neighborhood controls were visited at the designated location. Voluntary informed consent was obtained when enrolling controls and contacts. Follow-up and laboratory forms were completed and samples collected.

#### Laboratory and Data Management

The procedures for the laboratory and data management included sample transportation (Supplementary Figure 2), processing, storage, and documentation. This major area also included procedures for paper-based or electronic data capture and the management of data safety and entry. These procedures (Table 3) were monitored to ensure that they are conducted in accordance with the appropriate SOPs. An external microbiologist consultant was contracted to undertake the quality assurance process at the participating laboratories at least once every year. The consultant reviewed all the sample processing procedures on-site and flagged issues that required improvement and follow-up. The tool used to monitor and document findings (Supplementary Figure 3) was adapted from the International Organization for Standardization (standard 15189) [11].

A checklist for each procedure was developed and used to guide the monitoring (Table 3). The list details activities that at a minimum were observed during each visit regarding every aspect of the study, with the core activities acting as a guide (Figure 2). Activities were considered satisfactory if implemented according to the protocol and unsatisfactory if they required improvement.

### Central Monitoring of SETA Databases at IVI

Monitoring of SETA databases at IVI ensured the completeness, consistency, and quality of data recorded in databases. The data team checked all data from the field sites, both paper-based data collection and electronic data collection. A report for each site was generated for identified study indicators based on the objectives (Table 2) and shared with the principal investigator(s) (PI) at each specific site on a monthly basis. The report generated counts and proportions of indicators based on the study objectives. These included the number of patients screened, enrolled, and sampled, results of tests done, and contacts and controls under follow-up (Table 2). The report also outlined queries that needed to be addressed by the study team.

The observations made in the field complement the monitoring of databases, providing a comprehensive assessment of the surveillance performance and implementation of harmonized procedures at each site. All monitoring tools were subjected to pilot test at each site. This step was performed to assess and improve the tools and also to identify the unique cultural and geographical context of each site. These characteristics were considered during the implementation of the surveillance monitoring procedures.

### Implementation of Monitoring

Site monitoring visits were scheduled at least twice per year by dedicated IVI SETA staff; additional visits were scheduled when necessary. An agenda based on the core activities (Figure 1) was sent to the PI of each country a month in advance of the visit to provide flexibility for the necessary logistical planning or to modify the agenda to accommodate unique issues in each site. Upon arrival at the respective site, a brief meeting was held with the PI and members of the study team to finalize the agenda.

A process audit of the relevant procedures for each core activity was observed, documented, and was assessed in and outside the healthcare facilities, using the protocol and specific SOPs. A full day was spent in each healthcare facility to observe all study procedures and their implementation by study staff, starting with eligible patients identification, to transportation of samples to the laboratory for processing. Study documentation, including screening and enrollment logs, study forms (5%–10%) of newly collected forms since the last visit randomly were selected, and all informed consent forms, were reviewed for completeness and accuracy. Key procedures such as phlebotomy and blood culture were observed to verify whether they were being implemented per SOPs.

In the study laboratories, the processing of the samples was observed from the time they were received and processed/tested until point of storing. Accompanying documentation was also reviewed for accuracy and completeness. The forms reviewed in the laboratory included laboratory forms, samples transfer logs, samples logs documenting the performed assays, and the corresponding results.

For sites using paper data capture, dual data entry, tracked error correction, data protection, and dual password entry to access the computer were monitored. Secure storage of the complete forms was checked to determine whether the site is lockable and only accessible by key study staff. In sites capturing data electronically on a digital platform designed for the study called SETACollect, real-time data entry was observed.

Follow-up procedures, including control identification and enrollment and the sampling and packing of samples collected, were observed and documented. In instances when it was not possible to observe a particular procedure (e.g., unavailability of a case for follow-up), the activity was scheduled for a subsequent visit.

For each visit, a comprehensive report was written and shared with the respective PI. The report included the objectives of the visit, a list of personnel encountered at site, an executive summary, findings during the visit, and actions for immediate implementation and long-term follow-up. Issues requiring immediate attention were addressed with the study staff. Other corrective measures were highlighted to the in-country team during debrief at the end of the monitoring visit. An action plan (Supplementary Figure 4) was developed and discussed with the SETA team, especially the country focal point, requiring their support for implementation and follow-up. The action plan acted as a guide to track the progress of the recommendations made following each monitoring visit.

### External Monitoring

Additional monitoring was performed by the Scientific Advisory Process for Optimal Research on Typhoid burden of disease (SAPORT), on behalf of the Bill & Melinda Gates Foundation (BMGF). Elements monitored by SAPORT included project management and staffing, data management, enrollment of patients and controls, sample collection and transport, laboratory quality assurance/control, and follow-up visits. SAPORT, with a Secretariat at the Emory Global Health Institute, contracted by BMGF, provided direct research oversight for SETA [10] and SEAP (Surveillance for Enteric Fever in Asia Project) [12]. The SAPORT group was comprised of experts in the field of global health, infectious diseases, epidemiology and microbiology, biostatistics and modeling, and policy. They were identified through consultation of the SETA and SEAP PIs and the chairperson of the SAPORT group identified by BMGF. Part of their activities included yearly formal site monitoring visits and review of quarterly monitoring reports with key project indicators, and annual meetings with network PIs (SETA and SEAP) to deliberate on findings from the visits. These SAPORT activities ensured that surveillance and research were streamlined and harmonized between SETA and SEAP.

### Impact and Preliminary Results

Complete monitoring results will be published at the end of the study, but preliminary findings show the value of SETA monitoring and evaluation. One example is summarized here. The proportion of contaminated blood cultures is one of the data indicators used to measure the impact of monitoring. This indicator is important because the standard method for confirming typhoid fever in SETA is blood culture, and contamination in blood culture indicates whether blood culture collection procedures are being implemented according to SOPs.

**Figure 3.**
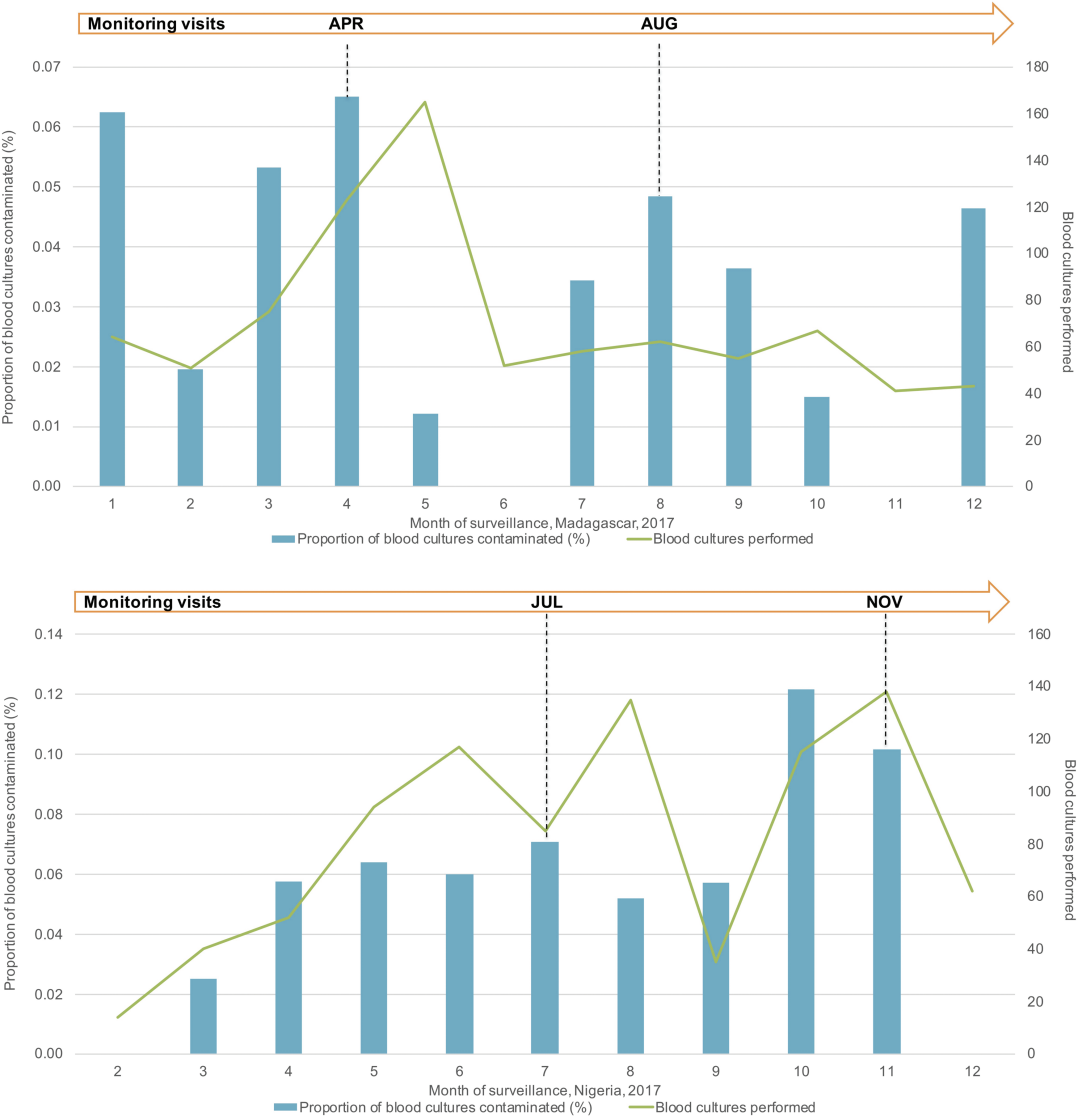
Proportion of blood cultures contaminated by month during 2017 in two SETA sites. Abbreviations: APR, April; AUG, August; JUL, July; NOV, November.

Figure 3 shows the proportion of blood cultures contaminated by month during 2017 in two SETA sites. The graph shows that contamination decreases after a monitoring visit, suggesting that phlebotomy techniques improve following support of on-site monitoring activities. However, specific events can trigger an increase in blood culture contamination. We observed such an increase in Nigeria when the trained study phlebotomist took leave; the proportion of contaminated blood cultures increased from 5.7% to 12.2% over two months (Figure 3). Results of a monitoring field visit included the implementation of additional training and close follow-up of phlebotomy procedures. The site elected to develop and use a phlebotomy checklist to identify areas where enhanced training and oversight were needed to improve the phlebotomy process. These activities resulted in a reduction in the proportion of contaminated blood cultures and lower dependence on a single study member for aseptic blood culture collection, providing an example of the positive impact of monitoring both data and field activities and the ongoing communication with the local study staff.

## CONCLUSIONS

Monitoring of epidemiological studies is important for data quality and to allow comparability across sites in a multicounty, multisite setting. Existing clinical trials and routine surveillance monitoring tools can be adapted for use in multicounty epidemiological studies. In implementing monitoring procedures, we observed that the environment and conditions of the countries participating in SETA vary as is likely to be the case for many multicountry, multisite epidemiological studies. Flexibility is required to navigate the dynamics of each study site and customized approaches have been implemented to adapt study procedures and overcome challenges in these individual conditions. Some of these challenges include political uncertainty and institutional and government bureaucracy. Nevertheless, rollout of core activities in line with the protocol and study SOPs required for data comparability across sites remained paramount, robust, and harmonized. The monitoring procedures and activities implemented have demonstrated value for the assessment of site performance and the identification of opportunities for improvement through on-site interaction with the site staff to ensure that recommendations made are addressed. It also allowed the creation of teams to identify solutions to the various challenges, making continuous feedback and team participation an important element in the success of monitoring activities. This underscores the value of considering routine monitoring of epidemiological studies.

## Supplementary Data

Supplementary materials are available at *Clinical Infectious Diseases* online. Consisting of data provided by the authors to benefit the reader, the posted materials are not copyedited and are the sole responsibility of the authors, so questions or comments should be addressed to the corresponding author.

ciz597_suppl_Supplemental_Figure_1

ciz597_suppl_Supplemental_Figure_2

ciz597_suppl_Supplemental_Figure_3

ciz597_suppl_Supplemental_Figure_4

ciz597_suppl_Supplemental_Figure_5

## Notes

***Acknowledgments.*** The authors thank the following site faculty and staff for their contributions to the monitoring and evaluation of the Severe Typhoid Fever in Africa program (SETA): Ini Adebiyi, Adegoke Adeoye, Sarah Agbi, Eunice Aroyewun, Halima Babalola, Aderemi Kehinde, Abigail Kogsey, and Oluwafemi Popoola, as well as the rest of the country team that facilitated the activity. The authors are also grateful to the International Vaccine Institute staff for their programmatic support, including Ji Hyun Han, Hyon Jin Jeon, and Soo-Young Kwon. Finally, the authors are grateful to Adwoa Bentsi-Enchill, World Health Organisation; Dennis Chao, Institute for Disease Modeling; Hope Johnson, GAVI; Gagandeep Kang, Translational Health Science and Technology Institute; Samuel Kariuki, Kenya Medical Research Institute; Eric Mintz, Centers for Disease Control and Prevention and, Jeffrey Stanaway, University of Washington, who were part of the SAPORT team.

***Financial support.*** This research was funded by the Bill & Melinda Gates Foundation [OPP1127988]. The International Vaccine Institute acknowledges its donors including the Republic of Korea and the Swedish International Development Cooperation Agency. This publication was made possible through a grant from the Bill & Melinda Gates Foundation [OPP1201031].

***Supplement sponsorship.*** This article was published as part of the supplement “Severe Typhoid Fever in Africa (SETA) Program” sponsored by the International Vaccine Institute.

***Potential conflicts of interest.*** All authors report no potential conflicts of interest. All authors have submitted the ICMJE Form for Disclosure of Potential Conflicts of Interest. Conflicts that the editors consider relevant to the content of the manuscript have been disclosed.
