# Supplementary figures and images for: The Monitoring and Evaluation of a Multicountry Surveillance Study, the Severe Typhoid Fever in Africa Program
Source: Clin Infect Dis. 2019 Oct 30;69(Suppl 6):S510–8. doi: 10.1093/cid/ciz597 (PMC6821296; doi:10.1093/cid/ciz597)

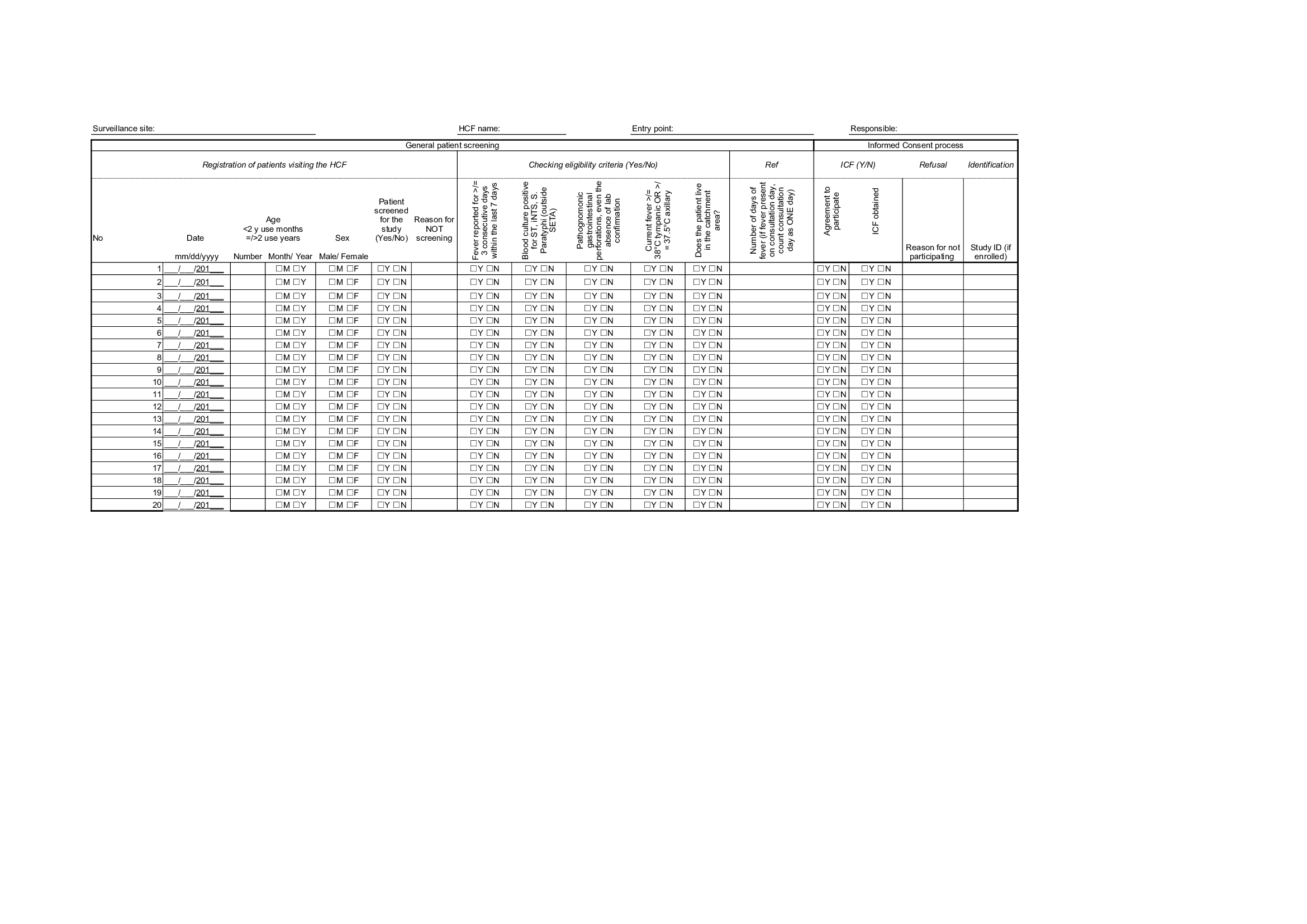

Supplement: ciz597_suppl_Supplemental_Figure_1 [file ciz597_suppl_supplemental_figure_1.png]

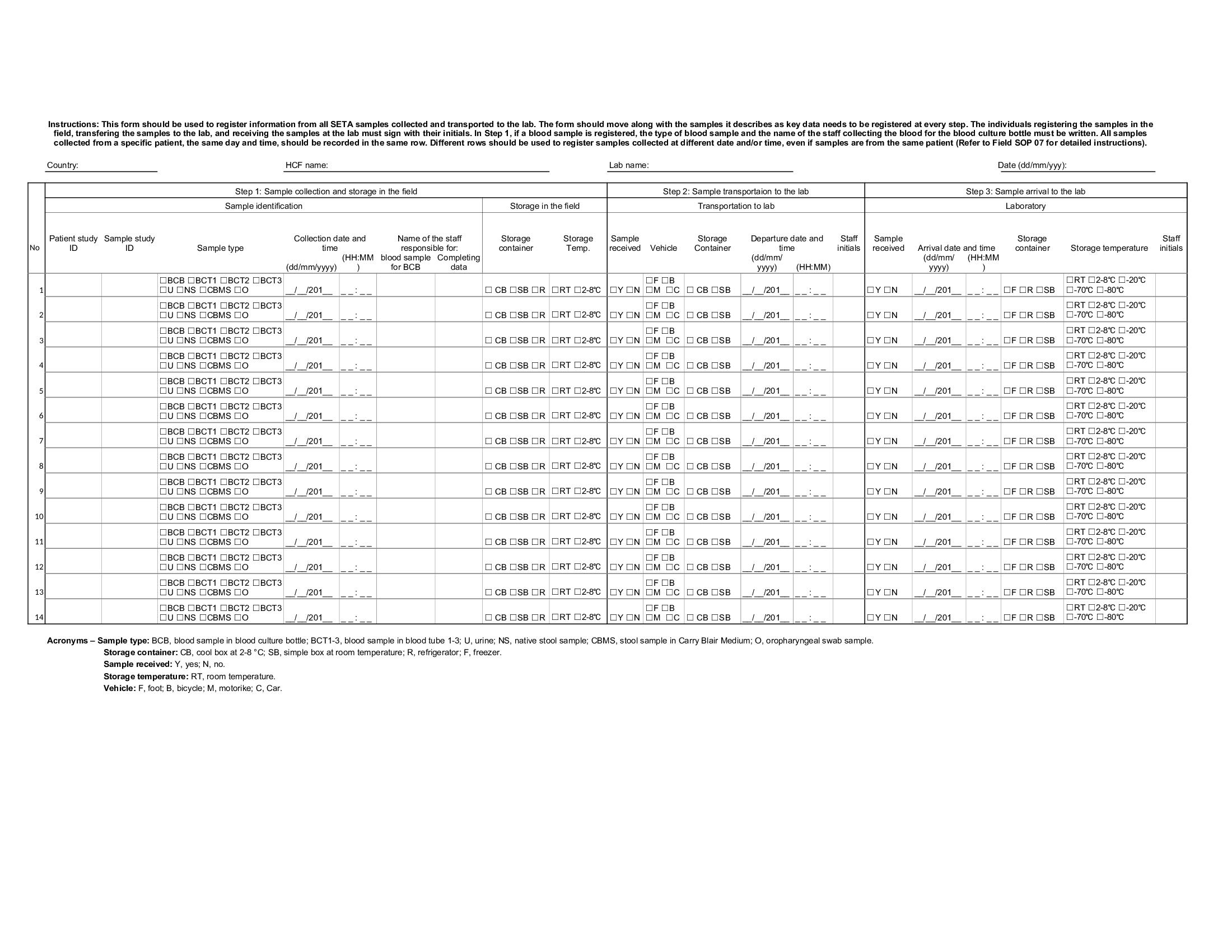

Supplement: ciz597_suppl_Supplemental_Figure_2 [file ciz597_suppl_supplemental_figure_2.png]

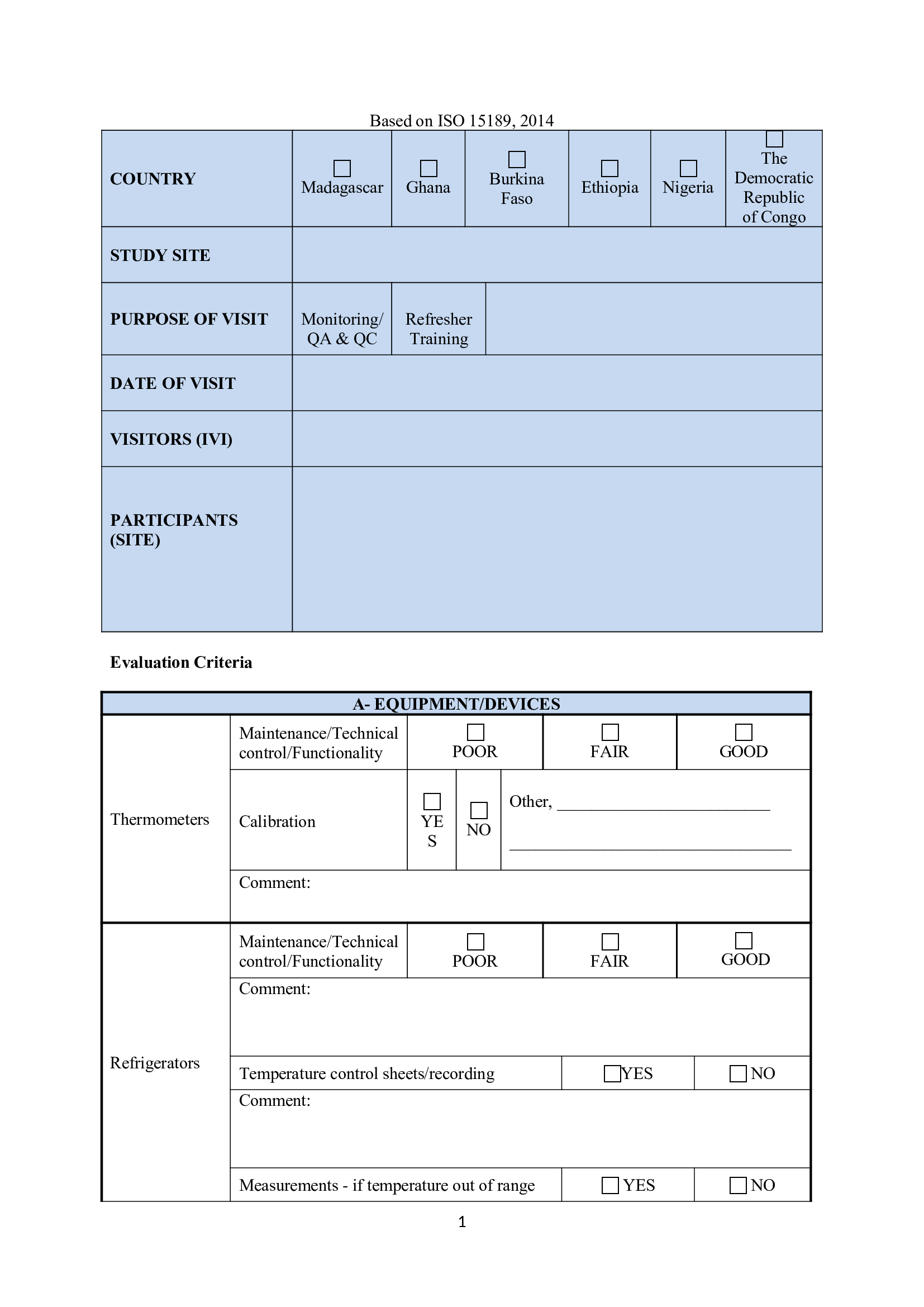

Supplement: ciz597_suppl_Supplemental_Figure_3 [file ciz597_suppl_supplemental_figure_3.png]

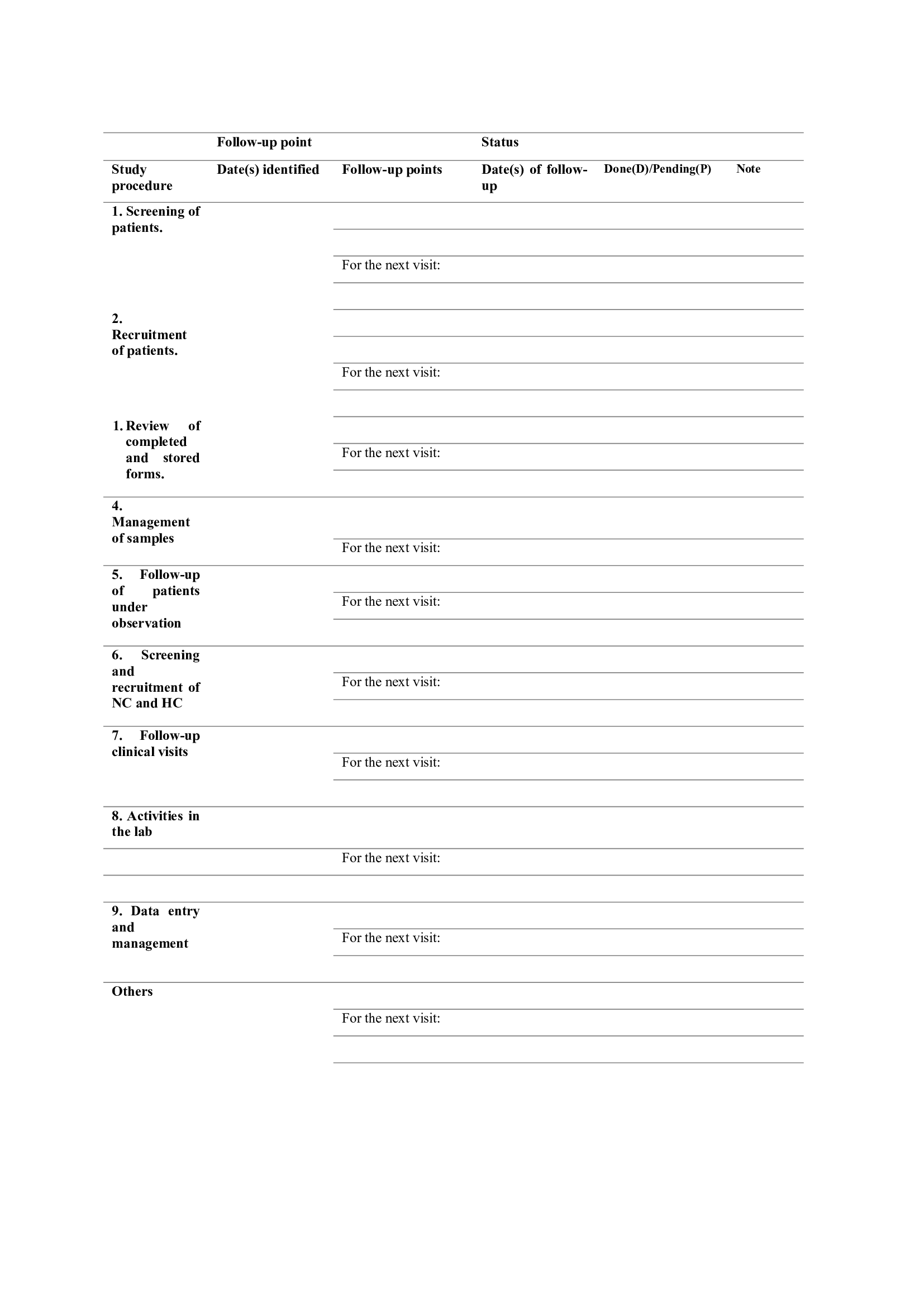

Supplement: ciz597_suppl_Supplemental_Figure_4 [file ciz597_suppl_supplemental_figure_4.png]

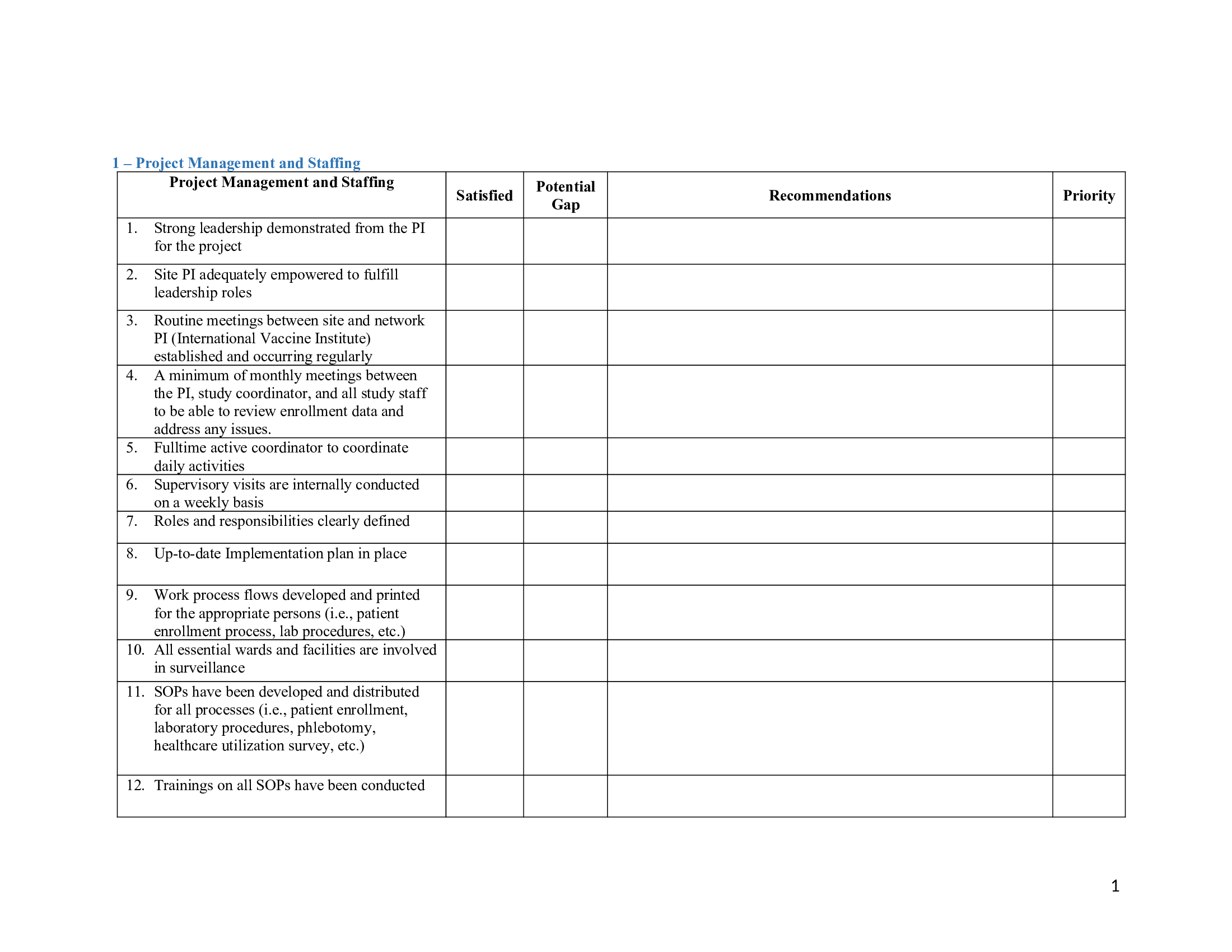

Supplement: ciz597_suppl_Supplemental_Figure_5 [file ciz597_suppl_supplemental_figure_5.png]
